# Echocardiography in Pregnancy: Part 2

**DOI:** 10.1007/s11886-016-0761-6

**Published:** 2016-07-25

**Authors:** Meena Narayanan, Uri Elkayam, Tasneem Z. Naqvi

**Affiliations:** 1 Division of Cardiology, Department of Medicine, Keck School of Medicine, University of Southern California, Los Angeles, CA USA; 2 Division of Cardiology, Department of Medicine, College of Medicine, Mayo Clinic, CK27, 13400 E Shea Blvd, Scottsdale, AZ 85259 USA

**Keywords:** Echocardiography, Pregnancy, Congenital heart disease, Valvular heart disease

## Abstract

The prevalence of pregnant women with cardiovascular heart disease is increasing. Transthoracic echocardiography is safe during pregnancy, and it is an important diagnostic tool in pregnant women with established heart disease in order to monitor ventricular and valvular anatomy and function. In addition, it can be used to delineate cardiac anatomy in complex congenital heart disease and help stratify maternal risk during pregnancy. This review will focus on the use of echocardiography in the diagnosis and management of pregnant women with common congenital lesions and with prosthetic valves.

## Introduction

Cardiovascular disease is a common cause of maternal mortality. Maternal risk during pregnancy can be assessed according to the modified World Health Organization (WHO) risk classification. In women who are WHO class I and II, risk is considered to be low to moderate and WHO Class III is considered high risk. In women who are WHO Class IV, pregnancy is considered a contraindication [1]. Transthoracic echocardiography is a safe and an important diagnostic tool in pregnant women with established heart disease in order to monitor cardiac condition and valvular function [2••]. Transesophageal echocardiography, although rarely required, is relatively safe during pregnancy and is particularly useful to delineate cardiac anatomy in patients with complex congenital heart disease. This review will focus on common congenital lesions and prosthetic valve disease and the advantages of echocardiography in the diagnosis and management of pregnant women.

## Echocardiography in Pregnant Women with Congenital Heart Disease

Due to advances in surgical repair, the number of women with congenital heart disease (CHD) who survive to childbearing age is increasing [3]. The main pathophysiologic mechanisms in pregnant women with CHD include volume overload, left-to-right shunts, pressure overload, and cyanotic right-to-left shunts [4]. Table 1 summarizes a list of CHD with increasing maternal risk. In general, pregnant women are able to tolerate left-to-right shunts without complications—which is not generally true for right-to-left shunts. For example, maternal mortality is similar to the general population with atrial septal defects compared to Eisenmenger’s syndrome, which carries an estimated risk between 25 and 50 % [5–7]. Regardless of pregnancy, echo interpretation is best achieved by a systematic interrogation of the atrial, ventricular, and great arterial segments, with determination of atrial and ventricular situs and connections of the atria to the ventricles and of the ventricles to the great arteries [8].

**Table 1:** Modified WHO classification of maternal cardiovascular risk in congenital heart disease

Low risk (WHO I)
Mild pulmonary stenosis
Small patent ductus arteriosus
Mitral valve prolapse with <mild mitral regurgitation
Successfully repaired atrial or ventricular septal defect, patent ductus arteriosus, anomalous pulmonary venous drainage
Intermediate risk (WHO II or III)
WHO II (if otherwise well and uncomplicated)
Unoperated atrial or ventricular septal defect
Repaired tetralogy of Fallot
WHO II–III (depending on individual)
Mild left ventricular impairment
Hypertrophic cardiomyopathy
Native or tissue valvular heart disease not considered WHO I or IV
Marfan Syndrome without aortic dilatation
Aorta <45 mm in aortic disease associated with bicuspid aortic valve
Repaired coarctation
High risk (WHO III)
Mechanical valve
Systemic right ventricle
Fontan circulation
Cyanotic heart disease (unrepaired)
Other complex congenital heart disease
Aortic dilatation 40–45 mm in Marfan syndrome
Aortic dilatation 45–50 mm in aortic disease associated with bicuspid aortic valve
Very high risk (WHO IV-pregnancy contraindicated)
Eisenmenger’s Syndrome Table
Marfan syndrome with aorta dilated >45 mm
Aortic dilatation >50 mm in aortic disease associated with bicuspid aortic valve
Native severe aortic coarctation
Severe systolic ventricular dysfunction (LVEF<30 %)
Previous peripartum cardiomyopathy with residual LV systolic dysfunction
Severe mitral stenosis
Severe aortic stenosis (with symptoms)

Modified from Thorne et al. [1], with permission from BMJ Publishing Group Ltd., and modified from Regitz-Zagrosek et al. [2••], with permission from Oxford University Press

## Specific Congenital Heart Defects

### Atrial Septal Defect

**Fig. 1:**
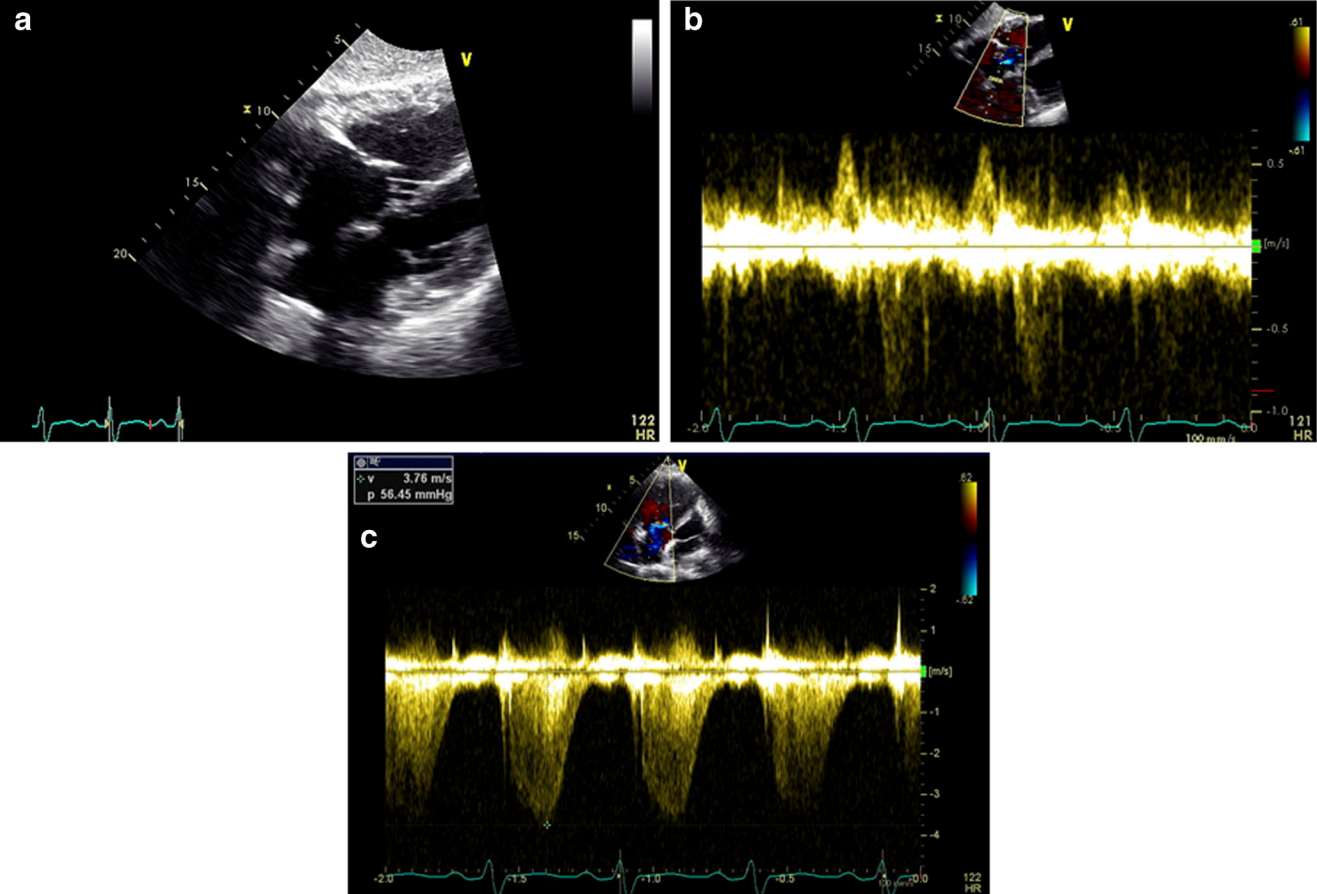
Incidental secundum atrial septal defect (ASD) measuring 1.9 × 1.5 cm seen in a pregnant patient who decompensated during delivery (**a**). Echo-Doppler revealed bidirectional shunting (**b**) and severely elevated peak right ventricular systolic pressure (RSVP), greater than 55 mm Hg (**c**). Patient was treated with intravenous diuretics and vasodilators. RVSP improved and patient was discharged home

Many women with atrial septal defects (ASD) may remain asymptomatic and are diagnosed initially during pregnancy. ASDs are generally associated with a left-to-right shunt across the septum. Shunt size can be estimated by 2D echo and pulse wave (PW) Doppler velocity measurements to estimate flow volume across the pulmonary and systemic circulations (expressed by the Qp/Qs ratio). Flow volume is the product of time velocity integral of blood flow and the cross-sectional area of the outflow tract. The degree of shunting depends on the defect size and right ventricular compliance. Usually, a typical ostium secundum ASD is well tolerated if there is no significant shunting [9]. Closure of a hemodynamically significant ASD should be performed before pregnancy due to risk of arrhythmia and thromboembolic complications [9, 10]. During pregnancy, increases in cardiac output and blood volume are counterbalanced by a decrease in pulmonary vascular resistance, with a resultant decrease in left-to-right shunting [11]. Echocardiography is useful in determining defect size, right atrial enlargement, right ventricular volume overload, and quantification of shunt size. Most importantly, pulmonary artery pressure can be estimated, which is a key determinant of maternal outcome (Fig. 1). In addition, contrast echocardiography for detection of significant shunting is not needed due to assessment by color Doppler and other features of a larger ASD or VSD. In general, saline contrast should be avoided during pregnancy to minimize any possible embolic risk [12]. Closure of an ASD is not indicated unless the mother is deteriorating; however, it can be performed during pregnancy if needed using transesophageal or intracardiac echo guidance [4].

## Ventricular Septal Defect

In contrast to ASDs, ventricular septal defects (VSD) are typically diagnosed and treated in childhood. Most detected VSDs in adulthood are small, perimembranous, and restrictive, with normal pulmonary artery pressure. On Doppler, a high velocity jet is seen from LV to RV with normal chamber dimensions and function [12]. These defects are well tolerated during pregnancy [4, 13]. However, in the presence of a large defect, left ventricular volume overload and RV hypertrophy occur. If pulmonary hypertension is present, shunt reversal can occur if there is significant reduction of the systemic blood pressure. In this case, serial echocardiography should be performed to evaluate for ventricular dysfunction and progression of pulmonary hypertension [11]. For patients with a history of corrected VSD, echocardiography should be performed prior to pregnancy to exclude presence of any residual VSD, to assess ventricular function and pulmonary artery systolic pressure (PASP). For patients with a prior history of primum ASD, echocardiography should be performed pre-pregnancy to evaluate for the presence of residual shunt, atrioventricular valve (AV) regurgitation, ventricular function, and PASP. Those with a significant shunt or with symptoms and with significant AV valve regurgitation should undergo corrective surgery prior to pregnancy provided pulmonary vascular resistance is normal [14].

## Patent Ductus Arteriosus

Similar to VSDs, left ventricular overload and pulmonary hypertension can be detected in pregnant women with a large patent ductus arteriosus (PDA). In a small PDA, chamber sizes are usually normal, although mild left atrial and left ventricular enlargement may be present. Careful color and spectral Doppler evaluation should be performed (in the parasternal and supasternal views). In significant PDA, main and branch pulmonary arteries are dilated, color aliasing is seen in systole and diastole in the main and branch pulmonary arteries depending on the location of PDA communication, and CW Doppler shows continuously increased systolic and diastolic velocities across the pulmonary arteries. Further evaluation should be performed in the suprasternal view to visualize the main pulmonary artery and right pulmonary artery, as well as the proximal descending aorta to visualize the PDA. If during pregnancy, left atrial and ventricular enlargements are more than expected and diastolic flow reversal is seen in the descending aorta in the absence of severe aortic regurgitation, a PDA should be suspected [12].

## Pulmonary Hypertension and Eisenmenger’s Syndrome

Pulmonary hypertension encompasses multiple conditions, including pulmonary arterial hypertension (idiopathic or heritable), pulmonary hypertension related to left heart disease, pulmonary hypertension related to lung disease and/or hypoxia, chronic thromboembolic disease, and pulmonary hypertension associated with congenital heart disease, with or without previous corrective surgery. A PASP of 50 mm Hg or greater than two-thirds the systemic pressure is considered high-risk during pregnancy. A high maternal mortality risk is reported (20–50 % in older series and 17–33 % in more recent papers) in patients with severe pulmonary arterial hypertension and Eisenmenger’s syndrome [6]. In Eisenmenger’s syndrome, the pulmonary vascular resistance is equal or higher than in the systemic circulation. Pulmonary hypertensive crisis and right heart failure are often the cause of maternal mortality in the peri-partum or postpartum period [15]. Even with the advancement of both obstetric and anesthetic managements, the maternal mortality still exceeds 25 % and significant functional deterioration occurs in the remaining patients [16]. Eighty percent of deaths occur between the 2nd and 30th postnatal day [17].

Once conception occurs in patients with Eisenmenger’s syndrome and severe pulmonary hypertension, interruption of pregnancy is still the best manipulation to be recommended. The primary anesthetic goal is to avoid any hemodynamic change that might increase the right-to-left shunt and thereby increase hypoxemia [18]. Treatment with sildenafil and l-arginine [19] or epoprostenol [20] has been reported to improve maternal and fetal outcome in isolated case reports. In addition, when HELLP syndrome (hemolysis, elevated liver enzymes, and low platelet count) occurs, more commonly seen in eclampsia and pre-eclampsia, it may cause an unfavorable outcome. The morbidity and mortality rates associated with HELLP syndrome have been reported to be as high as 25 % [21].

**Fig. 2:**
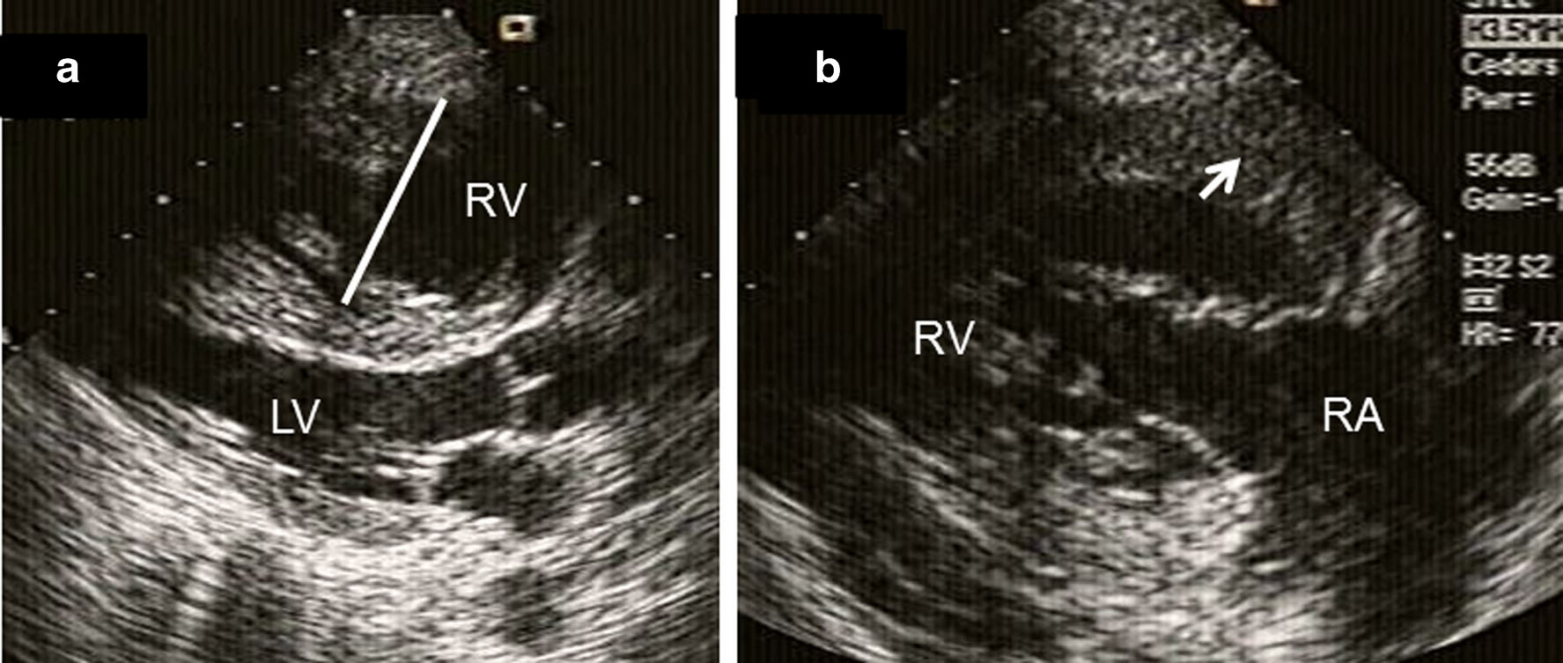
A 20-year-old Hispanic G_1_P_0_ was transferred with a diagnosis of primary pulmonary hypertension at 27-week gestation. There was evidence of polycythemia (Hgb 16 g, Hct 48 %), finger clubbing, and central cyanosis. Pulmonary artery pressure was 90 mm Hg and reduced to 80 mm Hg with inhaled nitric oxide. **a** Parasternal long-axis view showing severe right ventricular enlargement (*white line*), a small left ventricular cavity, and paradoxical IVS motion. **b** RV inflow view showing marked right ventricular hypertrophy (*white arrow*). *LV* left ventricle, *RA* right atrium, *RV* right ventricle

**Fig. 3:**
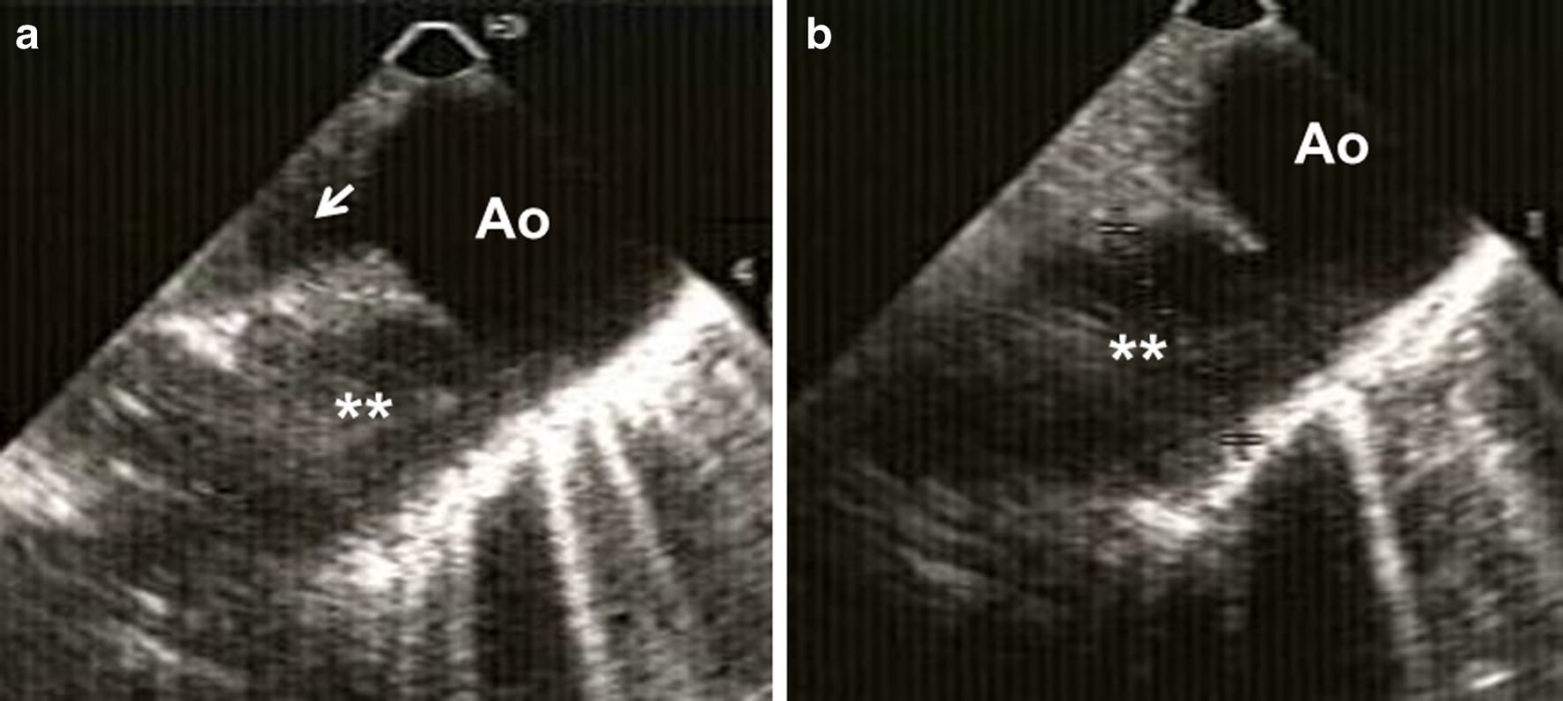
Due to fetal distress and maternal hypoxemia, a semi-emergent cesarean section was performed and a 28-week live female fetus was delivered. Intraoperative transesophageal echocardiographic images showing a large (1.8 cm in diameter) patent ductus arteriosus (*double white asterisks*) connecting with proximal thoracic aorta (*Ao*) (**a**). **b** shows aortic arch (*white arrowhead*). There was bidirectional flow across PDA. On postoperative day 2, patient underwent re-exploration of abdomen due to marked hypotension and marked cyanosis (reversal of shunt from PDA to aorta due to hypotension), and large amounts of clot were removed from the abdomen. Subsequently, the patient developed thrombocytopenia, disseminated intravascular coagulation, and elevated liver enzymes and died within 24 h

PASP may be difficult to detect in these patients due to an inadequate tricuspid regurgitant jet. Therefore, the presence of pulmonary hypertension can be inferred from evidence of a large, nonrestrictive cardiac communication, a short time-to-peak velocity on pulsed wave Doppler of the pulmonary artery, midsystolic notching of the pulmonary valve on M-mode echo, and abnormal ventricular septal motion. Increases of volume during pregnancy are poorly tolerated by the right ventricle, and the fall in systemic vascular resistance increases right-to-left shunting worsening cyanosis. Eisenmenger’s syndrome is associated with a high maternal risk (WHO Class IV) and high fetal risk, with high rates of spontaneous abortion and intrauterine growth retardation [2••]. Figures 2 and 3 are transthoracic echocardiographic and TEE images respectively in a 20-year-old Hispanic G_1_P_0_ who was transferred at 27-week gestation with a diagnosis of severe primary pulmonary hypertension. There was evidence of polycythemia (Hgb 16 g, Hct 48 %), finger clubbing, central cyanosis, right axis deviation, and RV hypertrophy on ECG. There was severe pulmonic insufficiency and PASP was 90 mm Hg. A PDA was diagnosed based on a saline contrast study that showed appearance of bubbles in the proximal thoracic aorta and aortic arch and not in the left ventricle. PASP reduced to 80 mm Hg on inhaled nitric oxide treatment. Due to fetal distress and maternal hypoxemia, a semi-emergent cesarean section was performed at 28-week gestation and a live female fetus was delivered. On postoperative day 2, patient underwent re-exploration of abdomen due to marked hypotension and marked cyanosis (reversal of shunt from PDA to aorta due to hypotension), and large amounts of clot were removed from the abdomen. Subsequently, the patient developed thrombocytopenia, disseminated intravascular coagulation, and elevated liver enzymes and died within 24 h.

## Pulmonic Stenosis

Many patients with pulmonic stenosis remain asymptomatic throughout early adulthood. Mild-to-moderate pulmonic stenosis is generally tolerated well during pregnancy. Severe pulmonic stenosis (peak Doppler gradient >64 mm Hg) can lead to right heart failure and arrhythmia and should be corrected before pregnancy [22–24]. However, secondary to the RV hypertrophy that occurs, women may be sensitive to the volume overload that occurs during pregnancy. A patent foramen ovale may be associated with PS, and in the setting of severe PS, there may be increased right-to-left shunting at the level of the atria [24].

## Left Ventricular Outflow Obstruction

This may be valvular, subvalvular (discrete membrane), or supravulvular. Congenital aortic stenosis (AS) is most commonly caused by a bicuspid aortic valve. Obstruction to aortic outflow results in LV hypertrophy and preload dependence, which may be problematic during labor, where significant volume shifts occur. Women with moderate or severe aortic stenosis with NHYA Class II symptoms are at risk for developing heart failure and premature labor. Echocardiography is useful in the assessment of LV function, aortic valve area, and transvalvular Doppler pressure gradients. Speckle tracking ultrasound can be used to assess longitudinal strain and LV twist. In a small study, LV twist in women with AS significantly increased during pregnancy compared to nonpregnant women [25]. Bicuspid aortic valves are associated with aortopathy and a higher risk of dissection. Women with bicuspid aortic valves should have their aortic root and ascending aorta evaluated by echocardiography before pregnancy. It is usually recommended that patients with bicuspid aortic valves and aortic root dilation >4.5 cm should avoid pregnancy [22].

## Coarctation of Aorta

Echocardiography should be performed in patients with a history of coarctation repair to evaluate for recurrence of coarctation and residual gradient and aortic aneurysm, as well as for the presence of a bicuspid aortic valve. Unrepaired coarctation of the aorta in pregnancy can cause increased risk of maternal hypertension and intrauterine growth retardation. Obtaining 2D scans of the coarctation may be difficult, but if a coarctation is seen will reveal a focal narrowing of the thoracic aorta distal to the left subclavian artery and turbulent flow on color Doppler. CW Doppler shows a high systolic velocity with a continued gradient in diastole. Due to the aortopathy risk associated with coarctation, there is an increased risk of aortic dissection during pregnancy. Additionally, aneurysm in the area of prior surgical repair can be seen in patients with coarctation. Both transthoracic and transesophageal echocardiography can be used to detect re-coarctation and evaluate pressure gradients across the thoracic aorta, or aneurysm complications. These patients represent a higher risk group and consideration of aneurysm repair before pregnancy may be warranted. In some cases, echocardiographic evaluation may be insufficient for full evaluation of aortic aneurysms, and other imaging modalities, such as magnetic resonance imaging or computed tomography, may be needed [26].

## Marfan’s Syndrome

Similar to bicuspid aortic valve and coarctation of the aorta, Marfan’s syndrome is associated with aortopathy. Women with Marfan’s syndrome in whom cardiovascular involvement is minor and the aortic root diameter is <40 mm tolerate pregnancy well. Conversely, patients with significant aortic dilation may experience more complications. In these patients, echocardiography should be done at 6–8-week intervals throughout the pregnancy until 6 months postpartum [27].

## Ebstein’s Anomaly

Ebstein’s anomaly is a rare condition in which the tricuspid valve is more apically displaced, causing “atrialization” of the right ventricle. A 20 mm or 8 mm/m distance between the septal tricuspid and anterior mitral leaflet is diagnostic [28]. This causes the right atrium to be large and the morphologic right ventricle to be small. It is often associated with patent foramen ovale or ostium secundum ASD defects. Paradoxical emboli and hypoxemia may occur secondary to reversal of shunt due to increased right heart pressure. RV failure can occur secondary to the increase in cardiac output and blood volume during pregnancy [11]. Echocardiography can be used to estimate the degree of tricuspid regurgitation and RV function. Tricuspid regurgitation is usually moderate-to-severe, and color Doppler can detect multiple jets from fenestrations of the leaflets [8]. In general, women with Ebstein’s anomaly without cyanosis and heart failure symptoms tolerate pregnancy well [2••]. In women with severe symptomatic tricuspid regurgitation or heart failure symptoms, tricuspid valve repair should be performed before pregnancy.

## Tetralogy of Fallot

**Fig. 4:**
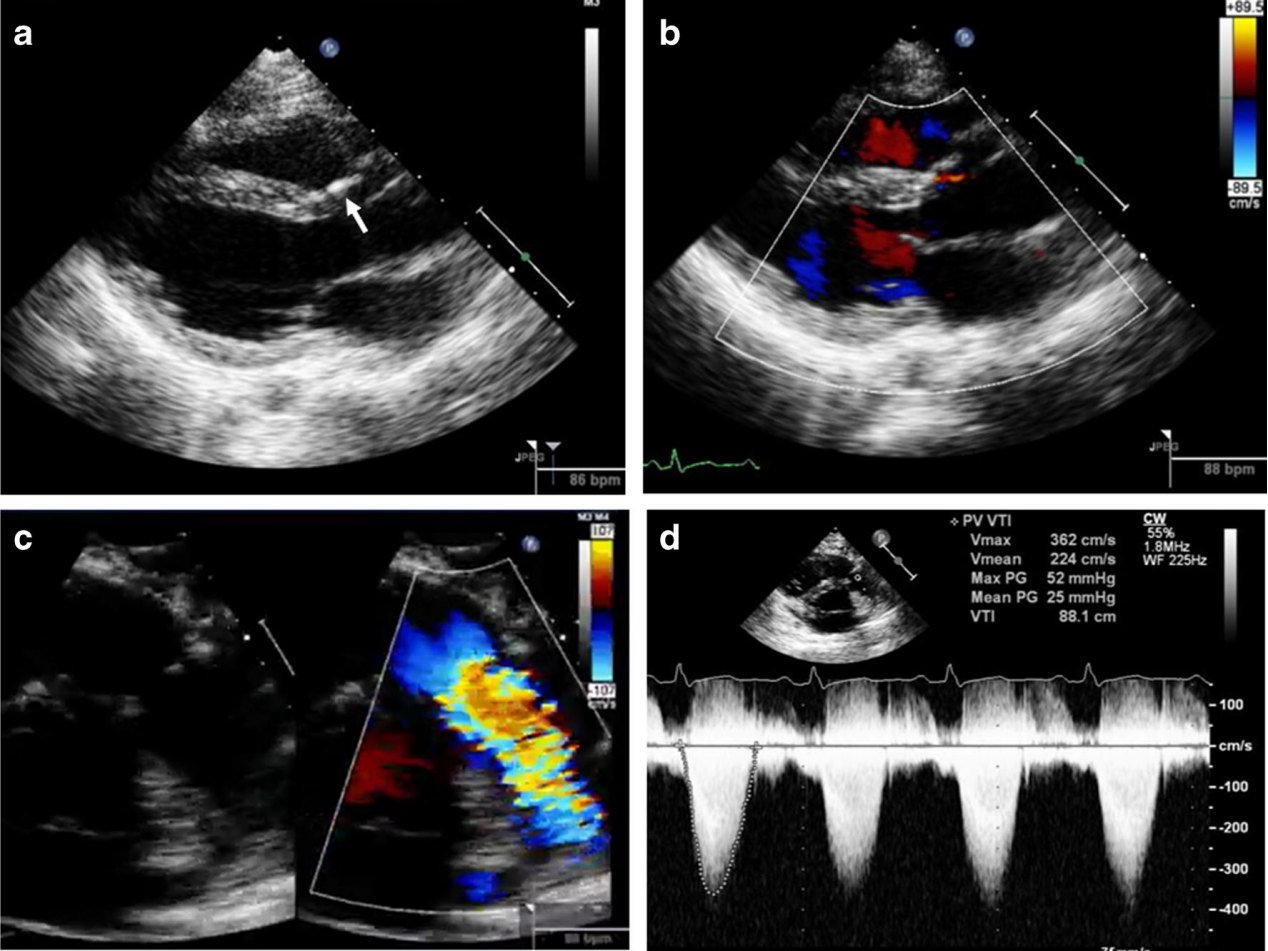
Echocardiographic images in a 24-year-old female G1 at 23 weeks with tetralogy of Fallot and surgeries at ages 3, 6, and 9 with VSD repair, pulmonic valve repair, and then replacement with a homograft. She also had a permanent pacemaker placement at age 14. NYHA class was II (able to walk two to three blocks at ground level and up 10 stairs). Images show the VSD patch in **a** (*white arrow*). There was no residual VSD flow and there was trace aortic regurgitation (**b**). Turbulent color Doppler flow across pulmonic prosthesis (**c**) with peak and mean gradients of 32 and 25 mm Hg, respectively, is shown (**d**). Patient is being closely followed in the OB cardiology clinic. The RV was mildly enlarged with paradoxical IVS motion and mildly reduced with RV systolic function. RV outflow tract was mildly dilated and there was intrinsic atrial rhythm (86–88 bpm) without RV pacing

Tetralogy of Fallot (ToF) is one of the most commonly repaired congenital lesions seen in adulthood [29–31]. Unoperated ToF is rare in adulthood and is associated with cyanosis and severe right ventricular hypertrophy. In these women, pregnancy is not advised because of poor maternal and fetal outcomes [32]. ToF repair includes relief of RV outflow obstruction with incision of the infundibular free wall, resection of obstructive muscle bundles, disruption of the pulmonary valve with partial or complete excision (which leads to pulmonic regurgitation in the majority of patients), and placement of an outflow patch, which often extends across the plane of the pulmonary valve into the main pulmonary artery. In some patients, a conduit between the RV and the pulmonary arteries is required to provide antegrade pulmonary blood flow. The ventricular septal defect is closed with a patch, a procedure that can impair tricuspid valve function. In individuals who have undergone repair, echocardiography should be performed before conception to evaluate RV size and RV function—particularly RV outflow tract function, residual pulmonic valve stenosis, pulmonic regurgitation severity, and systolic gradient and regurgitation of the pulmonic valve prosthesis, if present. Pulmonic regurgitation post-ToF surgery is well tolerated for many years until the third postoperative decade but thereafter may result in progressive RV enlargement and dysfunction. Other residual lesions include persistent RV outflow tract obstruction, residual VSD, aortic dilation, and aortic regurgitation. Due to RV enlargement and paradoxical septal motion or prior cardiopulmonary bypass, LV dysfunction may also occur [11, 33]. Elective pulmonary valve replacement is recommended for severe pulmonic regurgitation with RV enlargement [(RV end systolic volume index >80 ml/m^2^, end diastolic volume index >150 ml/m^2^, and RV ejection fraction less than 47 % on magnetic resonance imaging (MRI)] and exercise intolerance before pregnancy [2••, 34]. MRI is the gold standard to assess RV volumes and ejection fraction. Figure 4 shows echo images in a patient with ToF at 23-week gestation.

## Complete (D) Transposition of the Great Arteries

In 90 % of patients with complete D-transposition of the great arteries (D-CTGA), the aorta is anterior and to the right of the posterior and leftward pulmonary trunk, which can be identified on 2D echocardiography. The aorta is identified by the coronary arteries and the brachiocephalic arteries, while the pulmonary artery is identified by its major branches [8]. D-CTGA may be associated with VSD, subpulmonary outflow obstruction, and coarctation of the aorta. Most adults have undergone repair with an atrial switch (Mustard or Senning) or arterial switch (Jatene repair). The atrial switch entails a baffle that redirects systemic venous return to the anatomic LV and the pulmonary venous return to the anatomic RV. Echocardiography can evaluate complications such as baffle leaks or obstruction, vena caval or pulmonary venous obstruction, systemic atrioventricular valve (tricuspid) regurgitation, aortic regurgitation, and left and/or right ventricular dysfunction [8]. Arterial switch is the preferred surgical approach and commonly performed now. Complications include RV outflow tract obstruction, neoaortic and pulmonary regurgitation and dilation of the neoaortic root [32]. A comprehensive echocardiogram is essential before pregnancy and patients with a Mustard or Senning repair who should have monthly or bimonthly cardiac echocardiograms during pregnancy, to evaluate for development of pulmonary hypertension, severe tricuspid regurgitation, and RV dysfunction [2••]. Only a small series of patients with an arterial switch operation and pregnancy have been described so far, and the risk is low when the patients are in good clinical condition prior to conception [2••, 35].

## Congenitally Corrected L-Transposition of the Great Arteries

Congenitally corrected L-transposition of the great arteries (L-CTGA) is a rare form of CHD, in which there is atrioventricular and ventriculoarterial discordance. In this case, the RV serves as the systemic ventricle. The morphologic right ventricle is in the subaortic position and can be identified by the septal insertion of the left atrioventricular valve (tricupsid valve) that is more apically displaced compared to the right atrioventricular valve (mitral valve). The morphologic RV can also be identified by a moderator band. Associated defects include VSD, pulmonary stenosis, and Ebstein’s-like anomaly of the tricuspid valve. Outcomes of pregnancy depend on associated defects and systemic ventricular function. For both D- and L-TGA, women with NHYA Class III–IV and ventricular function <40 % and those with severe systemic atrioventricular valve regurgitation should be counseled against pregnancy. During pregnancy, evaluation for systemic atrioventricular valve regurgitation and systemic ventricular dysfunction is critical. Echocardiographic surveillance should be performed every 4 to 8 weeks [2••].

## Fontan Circulation

The Fontan operation is a palliative procedure to relieve cyanosis by directing systemic venous blood directly to the pulmonary arteries, in a patient in whom a single ventricle supplies both the systemic and pulmonary circulation. 2D echo and spectral Doppler assessment are essential in the evaluation of atriopulmonary or venopulmonary anastomosis and atrioventricular valve regurgitation. Complications which can be identified include cavoatrial shunting, atrial septal shunting, and obstruction to the surgically created anastomotic sites. Due to the increased venous flow in pregnancy and the fixed cardiac output of a Fontan circulation, women are at high risk of atrial arrhythmias and right-sided heart failure [11]. TEE has been recommended to rule out right atrial thrombi due to sluggish venous Fontan flow [36]. Pregnant women with Fontan circulation are considered high risk (WHO Class III), but pregnancy can be achieved safely depending on the physiology of the Fontan circuit [2••, 37, 38].

## Prosthetic Valve Disease

**Fig. 5:**
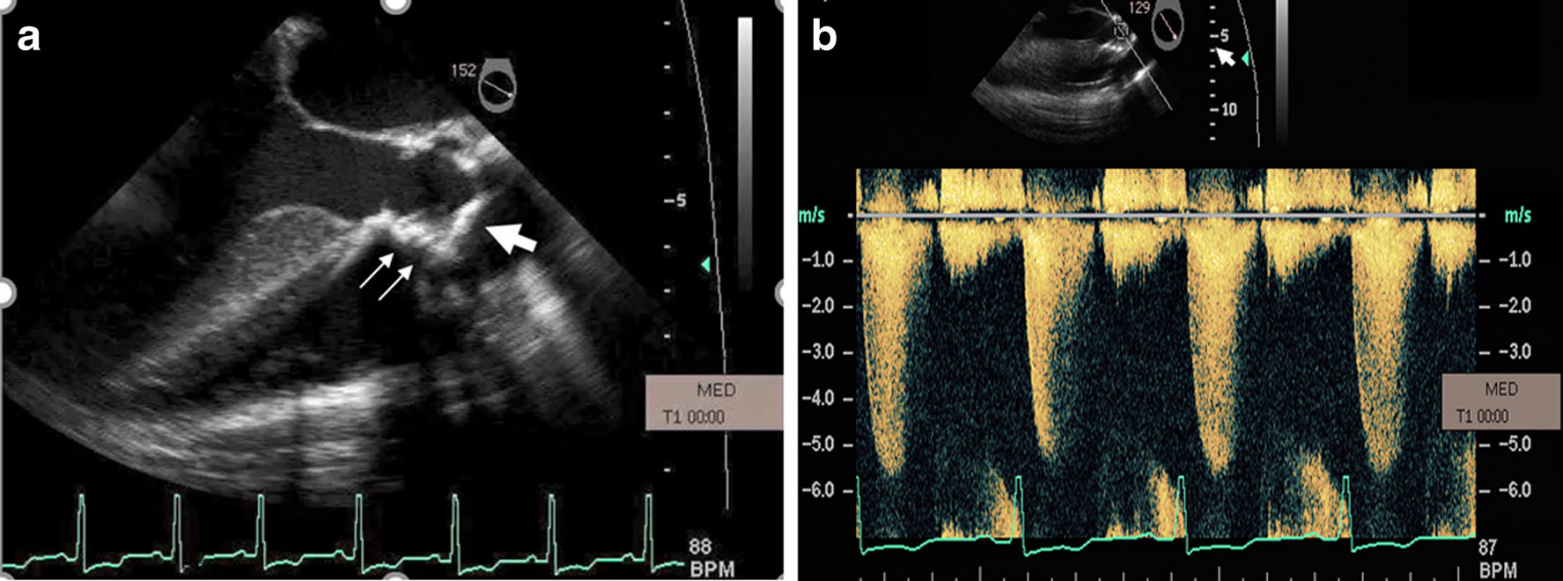
Thirty-two-year-old female with prior successful C-section 4 years ago developed hypotension postelective C-section at 37-week gestation. She had undergone bioprosthetic aortic valve replacement with a Carpentier-Edwards pericardial valve for severe aortic stenosis in a bicuspid aortic valve and aortic root enlargement with a pericardial patch due to a small aortic root 5 years ago. Due to dyspnea and an increase in mean prosthetic aortic valve gradient from 19 mm Hg pre-pregnancy to 51 mm Hg at 21-week gestation, an elective C-section was performed at 37 weeks. After extubation post-C-section, she developed pulmonary edema with a pulmonary capillary wedge pressure of 28 mm Hg and required pressor support. Transesophageal echocardiographic images showing calcified bioprosthetic aortic valve leaflets with restricted motion (*thick white arrow*) and prior pericardial patch (*thin white arrows*) in **a** and severely increased aortic valve gradient at 121 mm Hg in **b**

**Fig. 6:**
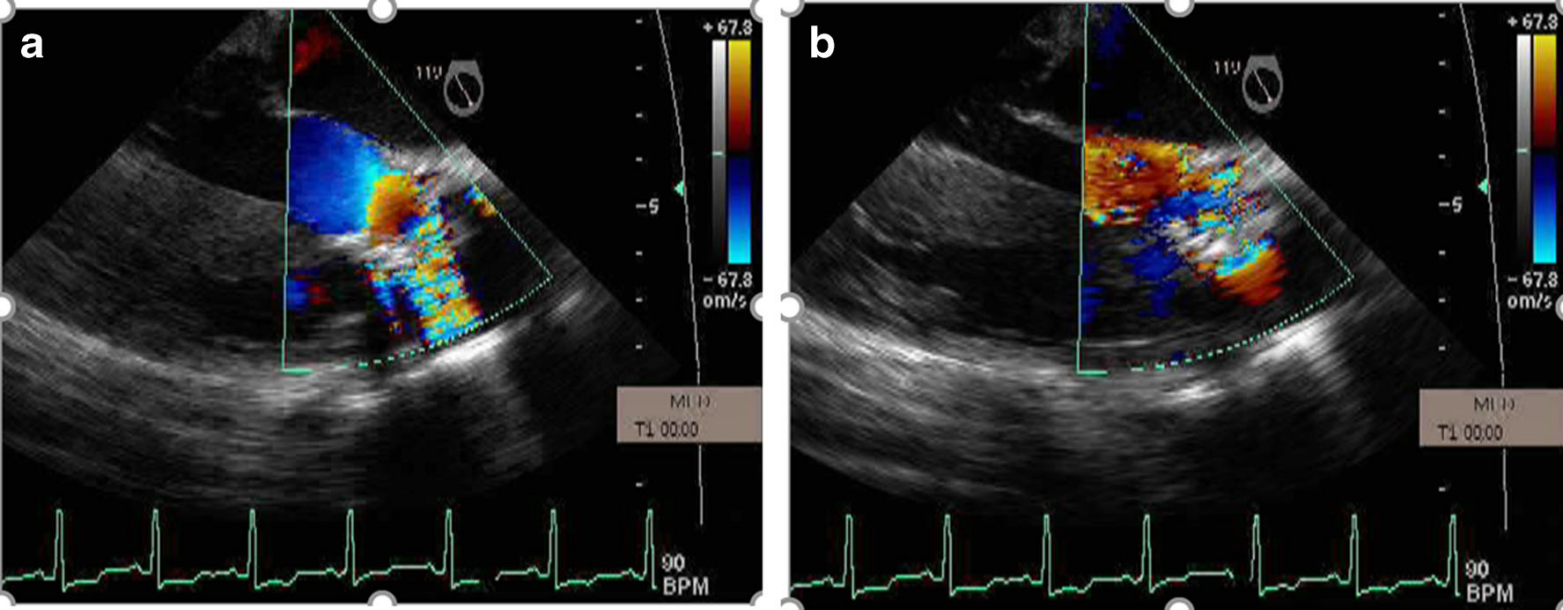
Transesophageal echocardiographic color Doppler images in the same patient as in Fig. 5 showing systolic Doppler flow convergence (PISA) toward the aortic valve at a normal aliasing velocity indicating a markedly increased gradient at the valve level from severe aortic stenosis in **a** and diastolic flow convergence (PISA) above the aortic valve at a normal aliasing velocity indicating severe aortic regurgitation in **b**. An emergent surgery was performed. Aortic leaflets were found fixed in mid open position. She underwent aortic valve replacement with a St. Jude Regent valve

Pregnancy outcomes are significantly affected by the presence of prosthetic heart valves (PHV). Prior to surgery, a detailed discussion regarding the type of valve needs to be considered. Specifics include durability of valve, thromboembolic risk, and the use of anticoagulation. Previously, it was reported that the rate of bioprosthetic valve degeneration in young patients (ages 16–39) was high, 50 % at 10 years and 90 % at 15 years [39]. Overall, about 50 % of women of childbearing age will require valve replacement 10 years after the initial operation with a bioprosthetic valve, and this risk is greatest when the bioprosthesis is in the mitral position [40]. There are conflicting data on whether there is an accelerated bioprosthetic valve degeneration during pregnancy [41–46] or whether there is just the natural deterioration of tissue valves [47, 48]. Bioprosthetic valves have a low thromboembolic risk, thus avoiding the need of anticoagulation and decreasing the risk of bleeding. Conversely, mechanical valves have great durability, but a higher risk of embolic events (3 to 5 %) and valve thrombosis (8 to 9 %). Overall, maternal mortality is higher with mechanical valves (4 to 8 %) compared to bioprosthetic valves (0 to 5 %) [12]. Despite the type of prosthesis, echocardiography is critical in identifying valve dysfunction and for management during pregnancy (Figs. 5 and 6).

Evaluation of prosthetic valves may be confounded by the normal hemodynamic changes that occur during pregnancy. Both the increased heart rate and stroke volume can alter Doppler evaluation of velocities and pressure gradients across the prosthetic valves. New murmurs can be secondary to a physiologic increase in flow; however, at least one echocardiogram should be performed during pregnancy and should be done with any development of symptoms. Similar to imaging native valves, prosthetic valve assessment includes color flow, PW and CW Doppler from multiple windows. Particular attention should be paid to occluded leaflet motion, presence or absence of echo densities attached to the sewing ring, cage, or struts, and the integrity of the sewing ring/annular interface [49]. These elements may be difficult to evaluate with standard 2D techniques because of shadowing and reverberation artifacts; therefore, 3D echo and TEE can be advantageous. Multiple off-axis views are often needed to evaluate valve morphology and function. Both transthoracic echo and TEE are used to evaluate for valve thrombosis when a pregnant woman with a mechanical valve presents with dyspnea and/or embolic events. Fluoroscopy can be helpful to determine the presence of prosthetic valve dysfunction but should be used in caution in pregnant women.

## Prosthesis-Patient Mismatch

Elevated transvalvular gradients can be seen secondary to intrinsic valve dysfunction but can also be seen in high flow states such as pregnancy, due to increased heart rate and blood volume. Therefore, careful evaluation of valve structure and comparison to prior echocardiograms are helpful to compare to postoperative (baseline) values. Prosthesis-patient mismatch (PPM) occurs when a valve is placed that is too small for a patient’s body size, resulting in a smaller area compared to the native valve [50]. In general, in patients with PPM, high gradients are already present in the early postoperative period. There have only been two case reports of patient prosthesis mismatch in pregnancy [51, 52]. PPM is defined by an indexed effective orifice area of <0.85 cm^2^/m^2^ for aortic prostheses and <1.2 cm^2^/m^2^ for mitral prostheses. Severe mismatch occurs when the indexed effective orifice area is <0.65 cm^2^/m^2^ for aortic prostheses and <0.9 cm^2^/m^2^ for mitral protheses [53]. It is important to distinguish PPM from pathologic obstruction (e, thrombus or pannus). With pathologic obstruction, valve leaflet motion and function will be abnormal.

## Acquired Prosthetic Valve Stenosis

**Fig. 7:**
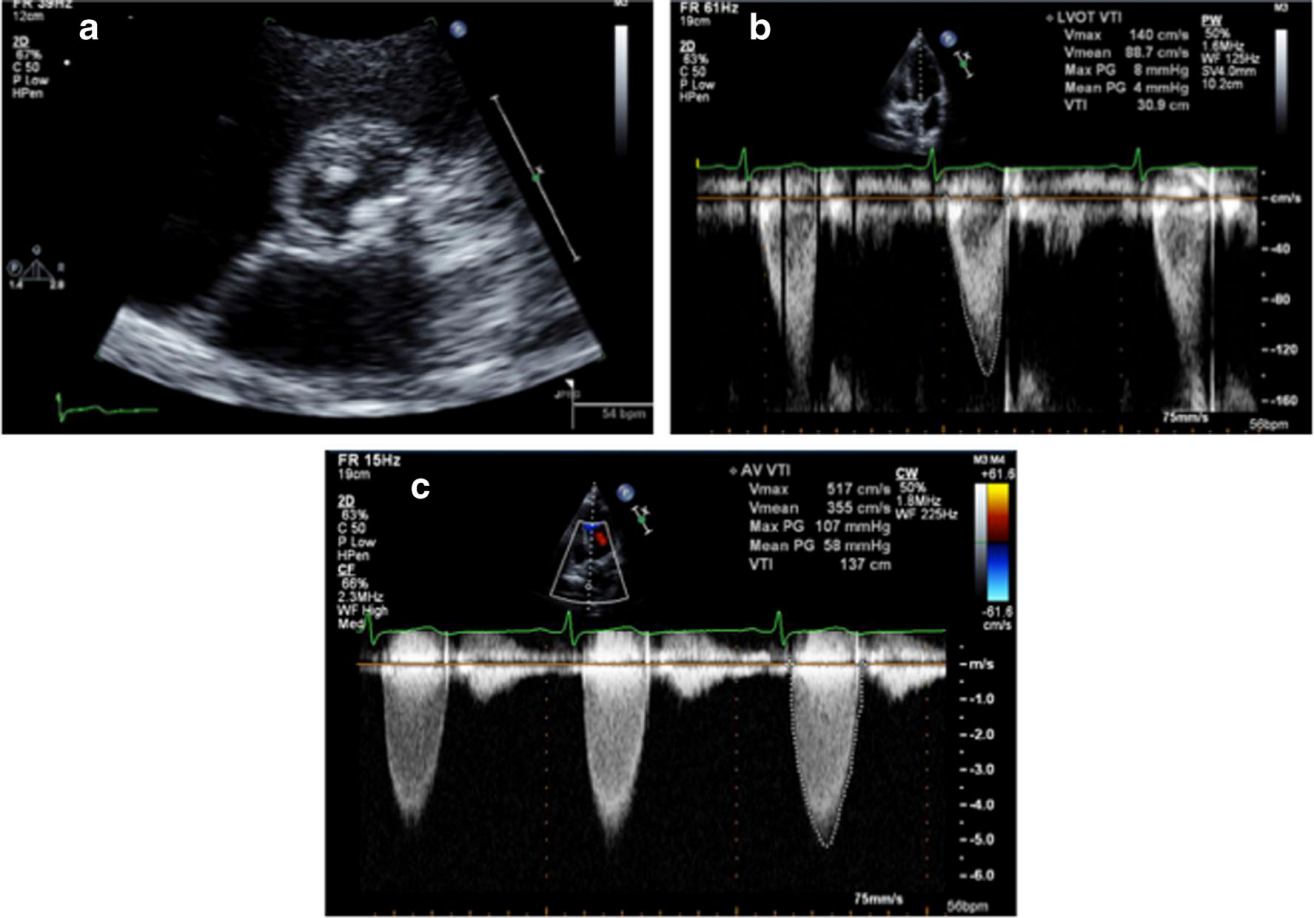
A 31-year-old pregnant woman with a history of St. Jude 21-mm bioprosthetic aortic valve replacement in 6 years ago (**a**, short-axis view) was found to have elevated gradients across her aortic valve on routine echocardiogram during her first trimester with an aortic valve area of 0.75 cm^2^ by continuity equation indicating severe aortic stenosis. Doppler velocity index—a ratio of PW Doppler subvalvular velocity (**b**) and the maximum CW Doppler velocity across aortic valve (**c**) was 0.25 indicating nonsevere aortic stenosis. Patient was followed closely throughout pregnancy and had normal vaginal delivery without any complication

Prosthetic valve stenosis can be secondary to thrombosis, endocarditis, pannus formation, or calcific degeneration of the valve leaflets. On serial echocardiograms, significant increases in gradients will be seen over time. Acceleration time (AT) can be used to help differentiate between aortic PPM vs acquired prosthetic stenosis. AT is measured from the onset of aortic flow to the peak velocity flow. PPM is associated with an AT <100 ms, compared to pathologic obstruction, in which AT is >100 ms [54]. The calculation of effective orifice area for both aortic and mitral valves requires measurement of the left ventricular outflow tract diameter, which can be challenging in the presence of an aortic valve prosthesis because of shadowing. Undermeasurement of LV outflow tract diameter will result in overestimation of aortic stenosis severity. In this situation, the Doppler velocity index (DVI) can be helpful. It is calculated as the ratio of the proximal flow velocity (or velocity time integral) in the LV outflow tract to the peak transprosthetic valve flow velocity (or velocity-time integral) (Fig. 7). For a mitral valve prosthesis, DVI is the ratio of the transprosthetic valve velocity to the LV outflow tract flow velocity. A normal DVI for an aortic prosthesis is >0.3 and for a mitral prosthesis is <2.2. A DVI of <0.25 reflects severe aortic stenosis [55].

## Prosthetic Valve Thrombosis

It is essential to establish if prosthetic valve dysfunction is secondary to pannus formation versus thrombus to determine appropriate treatment. In general, pannus is annular in location, gradually progressive, more common in the aortic position, and occurs in patients who have been on subtherapeutic anticoagulation [55]. Comparison to prior gradients and rate of increase in gradients is important. With valve thrombosis, increase in gradients and symptoms can be abrupt. In practice, both pannus and thrombus formation may be present together. When valve thrombosis is diagnosed, thrombolysis has been considered to be safe and effective during pregnancy in most patients; however, complications of bleeding and death have occurred [40]. Surgery is recommended if anticoagulation fails, in decompensated patients with obstructive thrombosis or with a large thrombus (>10 mm) and associated embolism [56]. However, surgery during pregnancy is associated with both increased maternal and fetal morbidity (24 and 9 %, respectively) and mortality (6 and 30 %, respectively) [57]. Ozkan demonstrated that TEE-guided, low-dose, slow infusion (6 h) of tissue-type plasminogen activator (tPA) was a safe and effective strategy for the treatment of prosthetic valve thrombosis in a small observational study. Both transthoracic echo and TEE were done prior to and 1 h postinfusion to assess for efficacy. After thrombolysis, mean valve area, peak, and mean gradients improved significantly, with no remaining visualized thrombus [56].

## Prosthetic Valve Regurgitation

Bioprosthetic valve regurgitation is secondary to leaflet degeneration. Mechanical valve prostheses are commonly designed with a bileaflet occluding mechanism. These valves have two small “washing jets” traveling between the inner edge of the sewing ring and the outer edge of the leaflets. In theory, these jets help to prevent thrombus formation. Pathologic transvalvular regurgitation is generally caused by thrombus or pannus formation causing incomplete closure of the discs—as opposed to paravaluvlar regurgitation, which is secondary to dehiscence of the valve ring. Quantification of regurgitation is similar to the assessment in native valves, except mechanical mitral prostheses that have significant shadowing, making color Doppler interpretation more difficult. If regurgitant jets are reliably visualized, then the standard methods for native valve assessment can be used to estimate the degree of severity. If transthoracic imaging is unsuccessful, TEE imaging may be indicated. Several parameters, such as mitral E velocity and Doppler velocity index, have been used to predict mitral regurgitation but have not been validated [49].

## Conclusion

The prevalence of pregnant women with cardiovascular heart disease is increasing. Echocardiographic assessment of biventricular function, valve function, quantification of shunts, and pulmonary pressure is essential. For mechanical and bioprosthetic valves, quantification of the degree of stenosis and regurgitation is similar to native valve assessment; however, gradients and the type of normal regurgitant jets vary depending on the type of valve. Echocardiography can be used for preconception counseling, to help advise termination of pregnancy in high-risk conditions and to decide mode of delivery. Lastly, both transthoracic echo and TEE have been used to monitor diagnostic and therapeutic procedures, such as treatment in valve thrombosis, percutaneous ASD closures, and balloon mitral valvotomy [12, 56, 58].
